# Traditional Chinese medicine auricular point acupressure for the relief of pain, fatigue, and gastrointestinal adverse reactions after the injection of novel coronavirus-19 vaccines: a structured summary of a study protocol for a multicentre, three-arm, single-blind, prospective randomized controlled trial

**DOI:** 10.1186/s13063-021-05138-3

**Published:** 2021-02-25

**Authors:** Qinwei Fu, Hui Xie, Li Zhou, Xinrong Li, Yang Liu, Min Liu, Chaoyu Wang, Xiaocen Wang, Zhiqiao Wang, Jinfan Tang, Huan Xiao, Zhiyong Xiao, Jing Zhou, Chengzhi Feng, Li Wang, Zhimin Ao, Xi Chen, Chang Su, Xuanyu Wu, Maolan Zhao, Sihan Hu, Hanwen Lin, Jiali Huang, Guo Xu, Qinxiu Zhang, Luyun Jiang

**Affiliations:** 1 Hospital of Chengdu university of Traditional Chinese Medicine, Chengdu university of Traditional Chinese Medicine, Chengdu, 610075 China; 2 Du Jiang Yan Medical Center, Du Jinag Yan, 611830 China; 3 Acupuncture and Tuina School, Chengdu University of Traditional Chinese Medicine, Chengdu, 610075 China; 4 Eye School of Chengdu University of Traditional Chinese Medicine, Chengdu, 610075 China; 5 School of Medical and Life Sciences, Chengdu University of Traditional Chinese Medicine, Chengdu, 611137 China

**Keywords:** COVID-19, Randomised controlled trial, protocol, Traditional Chinese medicine, Auricular point; Acupressure; Adverse reactions; COVID-19 vaccines

## Abstract

**Objectives:**

To investigate if traditional Chinese medicine (TCM) auricular point acupressure (APA) can alleviate and (or) reduce the pain (including injection site pain, headache, other muscle and joint pain), fatigue, and gastrointestinal adverse reactions (including nausea, vomiting, diarrhea), after the injection of novel coronavirus-19 vaccines (NCVs).

**Trial design:**

The study is designed as a multicentre, parallel-group, three-arm, single-blind, prospective, randomized (1:1:1 ratio) study.

**Participants:**

More than 360 participants will be recruited from healthy people who vaccinate NCVs in 5 community healthcare centres in the Sichuan province of China and 1 university hospital (Hospital of Chengdu University of Traditional Chinese Medicine).

Inclusion criteria:

①Vaccinators meets the conditions of NCVs injection and have no contraindications to it. The details shall be subject to the instructions of the NCVs used and the statement of medical institutions. The first dose of NCVs injection shall be completed within 24 hours from the time of injection to the time of enrolment;

②No redness, swelling, injury or infection of the skin or soft tissue of both ears, which is not suitable for APA;

③No history of alcohol and adhesive tape contact allergy;

④18-59 years old, regardless of gender;

⑤Those who were able to complete the questionnaire independently at the time of the first and second dose of NCVs and on the 3rd, 7th and 15th day after the first and second dose of NCVs respectively;

⑥Those who agree to participate in the trial and sign the informed consent, and can seriously abide by the precautions after the injection of NCVs and the requirements of traditional Chinese medicine auricular point plasters sticking and acupressure.

Exclusion criteria:

①Those who are not suitable to be vaccinated because they belong to the contraindication or cautious population;

②Those who have participated in other clinical trials within 4 weeks before the start of this study;

③No chronic/habitual/persistent headache, Muscle or joint pain, fatigue, diarrhea, nausea, retching or vomiting before the injection of NCVs, and no related diseases present (details of this item is listed in full protocol);

④Those who are in use or have received TCMAPA within 2 weeks before the trial;

⑤Pregnant or lactating women;

⑥Participants with other serious primary diseases and psychosis.

**Intervention and comparator:**

①Auricular point acupressure group: participants receive bilateral, symptom-specific TCMAPA in 5 auricular points (per side, 10 points bilateral) for 5 days, 3-4 times (about 1 min each time) of self-acupressure per day, after each NCVs injection (10 days in total).

②Sham auricular point acupressure group: participants receive bilateral, none symptom-specific, sham APA in 5 auricular points (per side, 10 points bilateral) for 5 days, 3-4 times (about 1 min each time) of self-acupressure per day, after each NCVs injection (10 days in total).

③Blank control group: Non-intervention blank control.

The Hebei medical device Co. Ltd, Hebei, China manufactured the auricular point sticking plasters.

**Main outcomes:**

Primary outcomes are all scores of visual analogue scale (VAS) based on subjective judgment of the participants included, including VAS score of pain at injection site, headache, muscle and joint pain, fatigue, nausea, retching, vomiting and diarrhea. Time points for outcomes above are the same: ①Immediately after first and second injection of the vaccine (Baseline assessment); ②Three days after first and second injection of the vaccine; ③Seven days after first and second injection of the vaccine; ④Fifteen days after first and second injection of the vaccine.

**Randomisation:**

Participants will be randomized in 1:1:1 ratio to each group by computerized random number generator, and independently in each sub-centre.

**Blinding (masking):**

Participants, information collectors and statistical evaluators will be blinded between APA group and sham APA group. No blinding in the control group.

**Numbers to be randomised (sample size):**

No less than 360 participants will be randomized in 1:1:1 ratio to each group.

**Trial Status:**

Protocol version 2.0 of February 3rd, 2021. Recruitment is expected to start on February 18th, 2021, and to finish on March 12th, 2021.

**Trial registration:**

This trial was registered in the China Clinical Trial Registry (ChiCTR) (ChiCTR2100043210) on 8th February, 2021.

**Full protocol:**

The full protocol is attached as an additional file, accessible from the Trials website (Additional file 1). In the interest in expediting dissemination of this material, the familiar formatting has been eliminated; this Letter serves as a summary of the key elements of the full protocol.

**Supplementary Information:**

The online version contains supplementary material available at 10.1186/s13063-021-05138-3.

## Supplementary Information

**Additional file 1.** Full study protocol.

## Data Availability

Not applicable.

