# Supplementary material for: Traditional Chinese medicine auricular point acupressure for the relief of pain, fatigue, and gastrointestinal adverse reactions after the injection of novel coronavirus-19 vaccines: a structured summary of a study protocol for a multicentre, three-arm, single-blind, prospective randomized controlled trial
Source: Trials. 2021 Feb 25;22:162. doi: 10.1186/s13063-021-05138-3 (PMC7905419; doi:10.1186/s13063-021-05138-3)
Supplement: Supplementary file 1 — Additional file 1. Full study protocol. [file 13063_2021_5138_MOESM1_ESM.pdf]

版本号：V2.0 版本日期：20210203

中医耳穴贴丸缓解新冠病毒疫苗注射后疼痛性、乏力性及胃肠道性不良反应疗效评价
临床研究方案

临床研究申办单位：成都中医药大学附属医院

临床研究负责人：张勤修、傅勤为

研究顾问指导专家：蒋路云、谢慧、周立、刘敏、李昕蓉、刘洋

临床研究参加单位：

成都市金牛区西安路社区卫生服务中心
成都市武侯区望江路社区卫生服务中心
成都市武侯区浆洗街社区卫生服务中心
成都市青羊区太升社区卫生服务中心
成都市青羊区草堂社区服务中心

目录

一、诚信申明 4
二、研究题目 4
三、研究计划书版本号 4
四、经费来源 4
五、研究事项执行流程图（表） 5
六、研究背景 5
七、研究目的 6
八、纳入和排除标准 6
8.1 纳入标准 6
8.2 排除标准 7
九、设计方案，设计模式图 8
9.1 试验总体流程图 8
9.2 试验细节（第一针疫苗注射后）流程图 9
9.3 耳穴贴丸操作流程图 10
十、样本量估算 11
十一、随机和隐蔽分组的方法 11
11.1 随机方法 11
11.2 隐蔽分组方法 11
十二、盲法 11
十三、测量指标 12
13.1 主要指标 12
13.1.1 第一、第二针疫苗注射前的基线指标 12
13.1.2 第一、第二针疫苗注射后的随访指标 14
13.2 次要指标 16
13.3 安全性指标 16
13.3.1 严重不良事件 17
13.3.2 不良反应 18
十四、对参试者有效性认定的定义 18
十五、不良事件和不良反应的定义、鉴定方法和管理制度 19
15.1 不良事件 19
15.2 严重不良事件 20
15.3 不良反应 22
15.4 试验中紧急破盲方法 22
十六、伦理考量 23
16.1 伦理委员会的选择 23
16.2 报批程序 23
16.3 知情同意过程 24
16.4 注册时间与注册机构 24
十七、参试者的征募 24
十八、参试者一般信息的收集 25
十九、基线指标和观测项目 25

19.1 第一、第二针疫苗注射前的基线指标......25
19.2 第一、第二针疫苗注射后的观测项目......27
19.3 次要指标......29
19.4 严重不良事件......29
19.5 不良反应......31
二十、标准操作规程......31
20.1 中医耳穴贴丸......31
20.2 耳穴选择......31
20.3 耳穴贴丸与按压操作方法......32
20.4 其它注意事项......32
二十一、统计分析方法......32
二十二、参试者管理制度......33
22.1 对新冠疫苗接种者进行研究前的宣教......33
22.2 对纳入参试者干预的管理......34
二十三、标本管理制度......34
二十四、药品和器材管理制度......34
二十五、数据管理制度......35
25.1 数据管理的目的......35
25.2 原始资料记录的制度......35
25.3 采集和录入（记录）人员......36
25.4 核对制度......36
二十六、数据安全与监察委员会的组成和工作职责......36
25.1 数据安全......36
25.2 监察委员会的组成和工作职责......37
二十七、研究团队......38
二十八、知识产权......39
二十九、发表计划......40
三十、原始数据共享计划......40
三十一、试验结束后对参试者的治疗和管理......40
参考文献......40

一、诚信申明

本研究保证操作严格按照卫计委 2016 年颁布实施的《涉及人的生物医学研究伦理审查办法》，国家中医药管理局 2010 年颁布实施的《中医药临床研究伦理审查管理规范》，WMA《赫尔辛基宣言》和 CIOMS《人体生物医学研究国际道德指南》等伦理原则，以及相关中医耳穴贴丸行业规范、预先商定好的临床试验计划实施，研究将通过多人记录、中心监督等方式保证数据记录的真实性。本研究为医院院内科研项目，无外来资助等，研究成员之间无利益冲突。

二、研究题目

①注册题目：中医耳穴贴丸缓解新冠病毒疫苗注射后疼痛性、 乏力性及胃肠道性不良反应疗效评价的临床研究；

②研究课题正式科学名称：中医耳穴贴丸缓解新型冠状病毒疫苗注射后疼痛性、 乏力性及胃肠道性不良反应：一项多中心、三臂、单盲的前瞻性随机对照试验。

三、研究计划书版本号

本版本为经成都中医药大学附属医院伦理委员会审核并提出修改意见的第二版（版本号：V2.0　版本日期：20210203）。第一版为（版本号：V1.0　版本日期：20210106）。

四、经费来源

本研究为成都中医药大学附属医院院内科研课题，经费来自课题，无外来经费。

五、研究事项执行流程图（表）

<table>
<tr><th colspan="2"></th><th>招募</th><th>分配</th><th colspan="4">第一针疫苗期间</th><th colspan="4">第二针疫苗期间</th></tr>
<tr><td colspan="2">时间点</td><td>-T1</td><td>0</td><td>T1*</td><td>T2*</td><td>T3*</td><td>T4*</td><td>T1*</td><td>T2*</td><td>T3*</td><td>T4*</td></tr>
<tr><td rowspan="4">入组事项</td><td>纳入/排除</td><td>X</td><td></td><td></td><td></td><td></td><td></td><td></td><td></td><td></td><td></td></tr>
<tr><td>知情同意书</td><td>X</td><td></td><td></td><td></td><td></td><td></td><td></td><td></td><td></td><td></td></tr>
<tr><td>随机分组</td><td></td><td>X</td><td></td><td></td><td></td><td></td><td></td><td></td><td></td><td></td></tr>
<tr><td>发放注意事项</td><td></td><td></td><td>X</td><td></td><td></td><td></td><td></td><td></td><td></td><td></td></tr>
<tr><td rowspan="3">干预措施</td><td>耳穴贴丸</td><td></td><td></td><td colspan="3">X———</td><td></td><td colspan="3">X———</td><td></td></tr>
<tr><td>假耳穴贴丸</td><td></td><td></td><td colspan="3">X———</td><td></td><td colspan="3">X———</td><td></td></tr>
<tr><td>空白对照</td><td></td><td></td><td colspan="8">无干预空白对照</td></tr>
<tr><td rowspan="4">CRF 表填写</td><td>个人基本信息</td><td></td><td>X</td><td></td><td></td><td></td><td></td><td></td><td></td><td></td><td></td></tr>
<tr><td>主要结局指标*</td><td></td><td></td><td>X</td><td>X</td><td>X</td><td>X</td><td>X</td><td>X</td><td>X</td><td>X</td></tr>
<tr><td>附加结局指标*</td><td></td><td></td><td>X</td><td></td><td></td><td></td><td>X</td><td></td><td></td><td></td></tr>
<tr><td>干预不良反应*</td><td></td><td></td><td>X</td><td>X</td><td>X</td><td>X</td><td>X</td><td>X</td><td>X</td><td>X</td></tr>
</table>

注：T1*：第 1 或第 2 针疫苗注射后立即；T2*：第 1 或第 2 针疫苗注射后第 3 天；T3*：第 1 或第 2 针疫苗注射后第 7 天；T4*：第 1 或第 2 针疫苗注射后第 15 天；主要结局指标*：疫苗注射部位疼痛 VAS 评分、头痛 VAS 评分、肌肉关节疼痛 VAS 评分、疲乏/乏力感 VAS 评分、恶心/反胃感 VAS 评分、干呕 VAS 评分、呕吐 VAS 评分、腹泻 VAS 评分；附加结局指标*：接种者对疫苗接种后可能产生的不良反应的了解程度、接种者对疫苗接种后可能产生的不良反应的担忧程度；干预不良反应*：包括贴丸处异常疼痛、皮肤过敏及其它不良反应的开放性记录。

六、研究背景

当地时间 7 月 20 日，《柳叶刀》发布了两款新冠病毒疫苗（COVID-19）1 期/2 期临床试验的最新结果[1,2]。其中一款由中国工程院陈薇院士团队及康希诺生物公司（CanSino Biologics）共同研发，另一款由英国牛津大学和阿斯利康公司开发。从实验结果来看，两款疫苗均能实现对新冠病毒（SARS-CoV-2）的免疫反应，并且诱导了高效的 T 细胞免疫应答。

在我国团队带来的这项非复制型 5 型腺病毒（Ad5）新冠病毒疫苗临床 2 期试验中，508 位参与者分别接受了高剂量（1E11 病毒颗粒，n=253）、低剂量（5E10 病毒颗粒，n=129）疫苗以及安慰剂（n=126）注射。该研究的主要结果是评估 Ad5 载体 COVID-19 疫苗的免疫原性和安全性，并确定 3 期功效研究的疫苗剂量。除此之外，一些研究及国产新冠疫苗说明书也表明可能会出现一些接种后不良反应，如疫苗注射部位疼痛、疲乏/乏力、腹泻、头痛、发热、疼痛、恶心/反胃、干呕、呕吐（呕吐出呕吐物）、咳嗽。

中医耳穴贴丸是重要中医外治法之一，具有无创、简便、可刺激时间长、不良反应极少（如压痛、胶布过敏）等特点[3-6]。迄今为止，中医耳穴贴丸疗法治疗的病种已达 200 余种，涉及内、外、妇、儿、五官、皮肤、骨伤等各方面[7-15]；不仅对某些功能性、变态反应性及炎症性疾病有较好疗效，也对部分器质性病变，以及某些疑难杂症也具有一定疗效[16-24]。耳穴贴丸不良反应极少，偶有出现如局部皮肤过敏反应、压痛等，且多于停止干预后消失[25-28]。

因此，本研究拟通过多中心随机对照临床试验的方式，为中医耳穴贴丸减轻、减少新型冠状病毒疫苗注射后疼痛性（包括注射部位疼痛、头痛、其它肌肉关节疼痛）、乏力性、及胃肠道性（包括恶心呕吐、腹泻）不良反应提供临床证据。

七、研究目的

研究中医耳穴贴丸是否能够减轻、减少新型冠状病毒疫苗注射后疼痛性（包括注射部位疼痛、头痛、其它肌肉关节疼痛）、乏力性、及胃肠道性（包括恶心呕吐、腹泻）不良反应以及其所能减轻的程度。

八、纳入和排除标准

8.1 纳入标准

①符合新冠疫苗注射条件，无新冠疫苗注射禁忌症，详情以所使用新冠疫苗说明书及医疗机构声明为准；完成第一针新冠疫苗注射且从注射后到入组时≤24 小时；

②无耳部皮肤或软组织红肿、损伤、感染等不适宜耳穴贴丸的情况；

③无酒精及胶布体表接触过敏史；

④18-59 周岁，性别不限；

⑤能够于注射第一、第二针疫苗时及分别于注射第一、第二针疫苗后第 3、7、15 天独立完成本研究调查问卷者；

⑥同意参加本次试验并签署知情同意书者；能够认真遵守新冠疫苗注射后注意事项及中医耳穴贴丸需知。

8.2 排除标准

①因属于新冠疫苗注射禁忌或慎用人群，而暂时不宜接种新冠疫苗者；

②本研究开始前 4 周内曾参加过其他临床试验；

③近 1 周内出现头痛≥3 次且其疼痛程度对日常生活造成影响；或参试者自述有以下头痛类疾病：如原发性头痛如偏头痛、紧张性头痛、颈源性头痛，及其它导致继发性头痛的疾病；或参试者有长期慢性头痛病史（＞3 个月），但未明确诊断或原因不明；

④近 1 周内出现全身其它部位肌肉或关节疼痛≥3 次且其疼痛程度对日常生活造成影响；或参试者自述现有肌肉或关节疼痛类疾病：如肩关节周围炎疼痛期、肌腱炎、肩袖损伤、腱鞘炎、骨滑囊炎、肌肉或关节损伤、风湿或类风湿性关节炎疼痛期，以及伴有疼痛的肌肉或关节劳损、颈椎病、椎间盘突出症；或参试者有长期慢性肌肉或关节疼痛病史（＞3 个月），但未明确诊断或原因不明；

⑤近 1 周内出现原因不明的明显疲乏/乏力感≥3 次，且疲乏/乏力程度对日常生活造成影响；或参试者自述有以下疲乏/乏力感相关疾病：如恶性肿瘤（癌症）、低血压、慢性疲劳综合征、贫血、肺源性心脏病、慢性阻塞性肺疾病、感冒、甲状腺功能减退症、低血糖、心衰、糖尿病等；或参试者有长期疲乏/乏力病史（＞3 个月），但未明确诊断或原因不明；

⑥近 1 周内出现原因不明的腹泻≥3 次且对日常生活造成影响；或参试者自述有以下腹泻相关疾病：如肠炎、甲亢、糖尿病、消化道恶性肿瘤、克隆病、肠结核、肠道真菌感染、慢性细菌性痢疾等；或参试者有长期腹泻病史（＞3 周），但未明确诊断或原因不明；

⑦近 1 周内出现原因不明的恶心/反胃、干呕或呕吐≥4 次且对日常生活造成影响；或参试者自述有以下恶心/反胃、干呕或呕吐相关疾病：如美尼尔氏综合征、前庭神经炎、胃炎、肠炎、胃食管反流病、肠梗阻、肠套叠、晕动病、甲亢、消化道恶性肿瘤等；或参试者有长期恶心/反胃、干呕或呕吐病史（＞1 月），但未明确诊断或原因不明；

⑧正在使用或本试验开始前 2 周内曾接受中医耳穴贴丸治疗者；

⑨孕妇或哺乳期妇女；

⑩合并有其它严重原发性疾病及精神病者。

九、设计方案，设计模式图

本研究为前瞻性、随机、三臂平行对照的探索性干预研究，由 1 家三甲医院（研究主办单位）及 5 家社区卫生服务中心的多位研究者参与。对中医耳穴贴丸组与假耳穴贴丸组的数据收集者、结局评估统计者及参试者施盲，空白对照组无盲法。

9.1 试验总体流程图

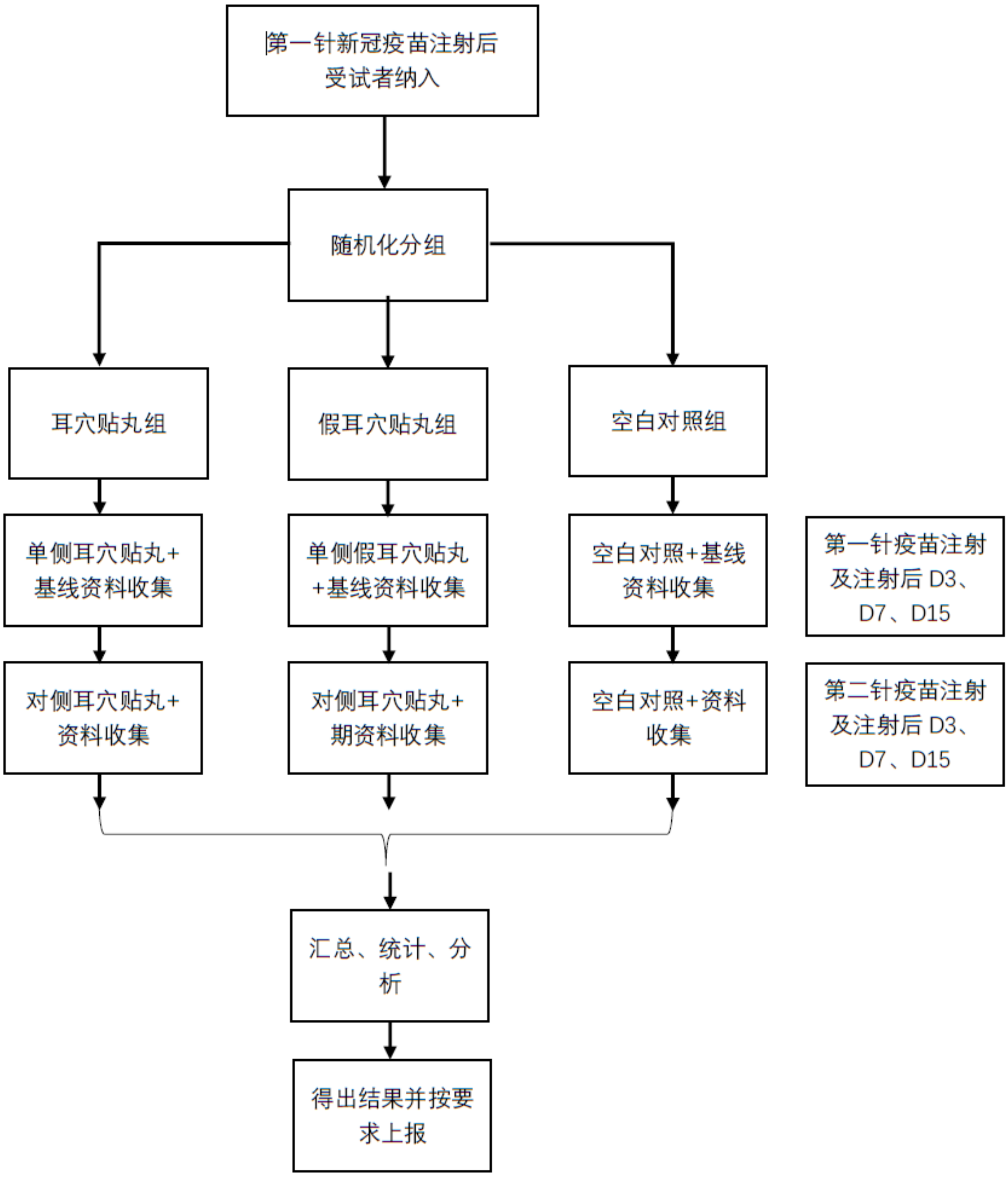

9.2 试验细节（第一针疫苗注射后）流程图

项目总体流程图（第一次疫苗注射后）

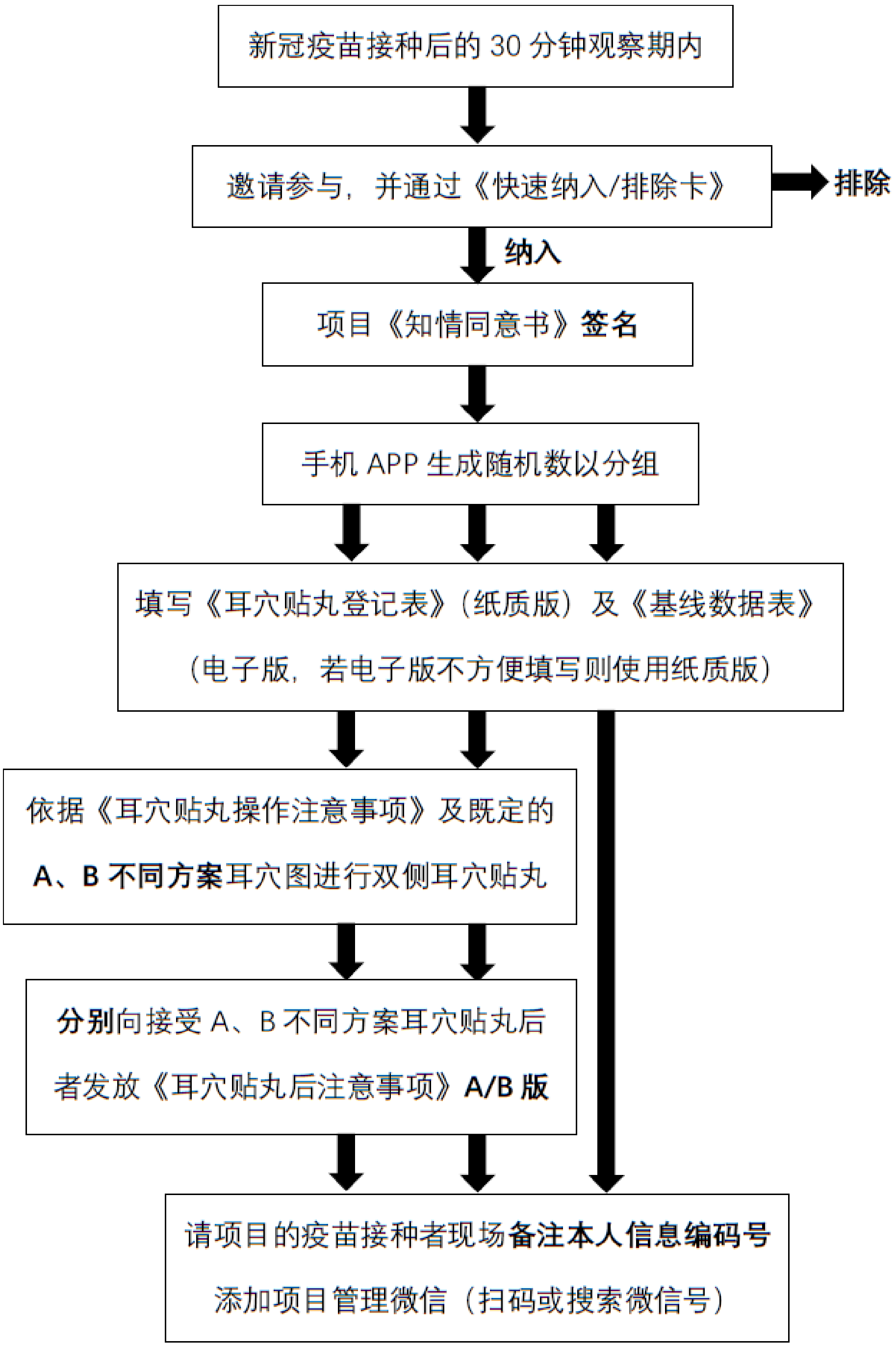

9.3 耳穴贴丸操作流程图

耳穴贴丸操作流程及注意事项

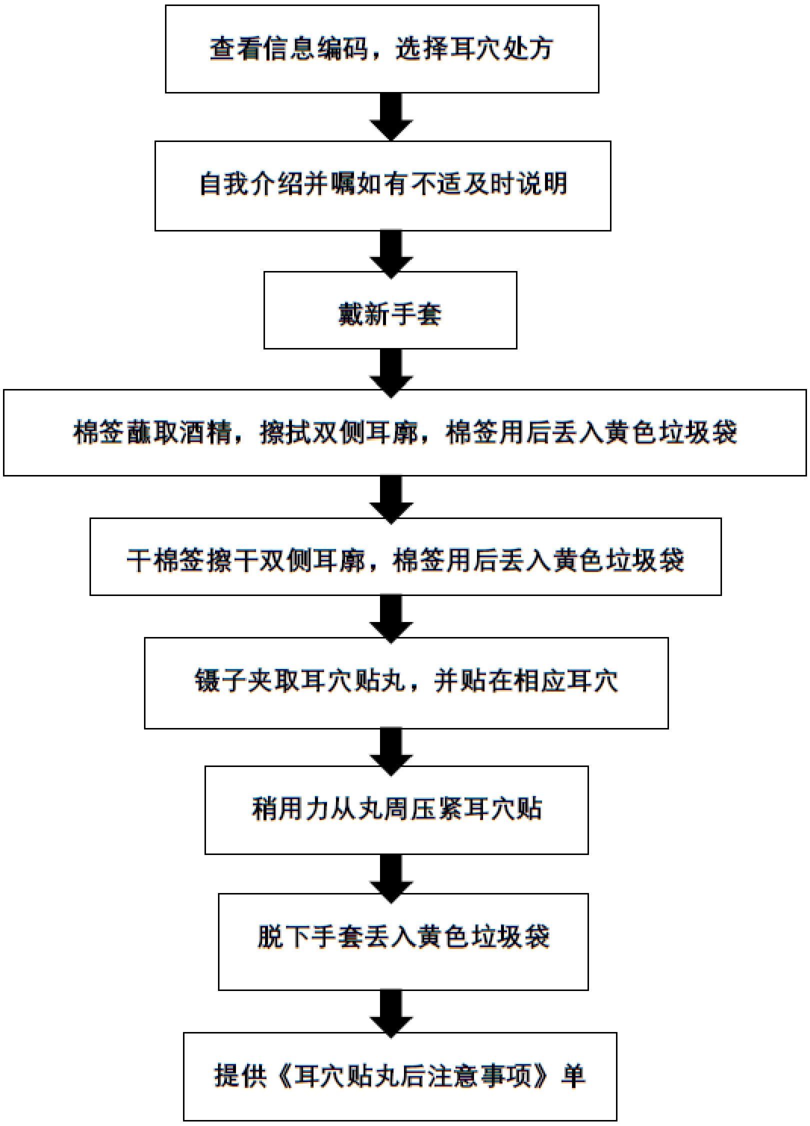

注意事项：

1.一人一手套，每位疫苗接种者进行耳穴贴丸时，及时更换一副新手套；

2.已接触皮肤的棉签禁止放回酒精瓶蘸取酒精；

3.贴丸完成后告诉接种者若日常按压耳穴贴时有局部胀感为正常得气感。

十、样本量估算

由于新冠疫苗注射的特殊性、紧急性，故本研究未进行预实验，并且无相关研究提供基线率及干预后拟提高率的数据。

因此，本临床试验拟按照 1:1:1 对照、20％富余的原则，总病例数选定为 360 例，中医耳穴贴丸组、中医假耳穴贴丸组与空白对照组各 120 例。应该完成全程观察病例数不低于 300 例。

鉴于目前新冠疫苗具体安排与进展的不确定性，各试验中心病例数分配以各中心实际新冠疫苗接种数为准，尽量在此基础上增加纳入人数。

十一、随机和隐蔽分组的方法

11.1 随机方法

在参试者纳入后，由组长单位指导使用计算机随机数字生成器，以各个分中心为独立样本进行三组的随机分配。每一个编码设有对应的应急信封。盲底由组长单位与本试验无关的人员保存。

11.2 隐蔽分组方法

在参试者被纳入研究时，每一位参试者都会被分配一独一无二的参试者信息代码。在经过随机分组后，代码即被记录完成。信息代码的生成者、记录者不参与耳穴贴丸、数据提取、结果分析等其它临床试验步骤，信息代码的解读规则被该生产者保密直至研究结束或因紧急情况而需要的揭盲。

十二、盲法

在参试者被纳入研究时，每一位参试者都会被分配一个一独一无二的参试者信息代码。在经过随机分组后，该代码即被记录。信息代码的生成者、记录者不参与耳穴贴丸、数据提取、结果分析等其它临床试验步骤。信息代码的解读规则被该生产者保密直至研究结束或因紧急情况而需要的揭盲。耳穴贴丸操作者在收到参试者的信息代码后，根据其中的分组代码选择相应的耳穴简图（无穴位名称）进行贴丸。

需要说明的是，可能有经验丰富的耳穴贴丸操作者能够仅仅凭借耳穴部位认出穴位，进而了解该耳穴的功效。尽管如此，考虑到耳穴贴丸这种中医外治法的特殊性，我们拟采用这种分配隐藏方法来尽量对耳穴贴丸操作者施盲，尽量减少可能的偏倚。

综上所述，尽管我们通过信息代码分配隐藏拟对耳穴贴丸操作者和信息采集及统计评价者施盲，但是考虑到可能有经验丰富的耳穴贴丸操作者能够仅仅凭借耳穴部位认出穴位进而了解该耳穴的功效，本研究被定义为中医耳穴贴丸组与中医假耳穴贴丸组之间的单盲（参试者、信息采集及统计评价者）更加谨慎。

十三、测量指标

13.1 主要指标

主要指标的测量时间为第一、第二针疫苗注射后立即及分别于注射第一、第二针疫苗后第 3、7、15 天，其测量方式皆为 VAS 数字模拟评分量表。其中第一、第二针疫苗注射后立即测量的结果分别为第一、第二针疫苗注射前的基线指标，其余为随访指标。具体如下：

13.1.1 第一、第二针疫苗注射前的基线指标

①疫苗注射部位疼痛

在疫苗注射前，我最近 3 天内有出现不明原因的疫苗注射部位疼痛

次数：约（　　　）次 或 每天断断续续发生（　　　）

严重程度：

0	1	2	3	4	5	6	7	8	9	10
无	有，轻微且尚能忍受			有一定疼痛，会影响工作、生活但尚能忍受			有，疼痛强烈，甚至影响食欲、睡眠			

②头痛

我最近 3 天内有出现不明原因的头痛

次数：约（　　　）次 或 每天断断续续发生（　　　）

严重程度：

0	1	2	3	4	5	6	7	8	9	10
无	有，轻微且尚能忍受			有一定疼痛，会影响睡眠但尚能忍受			有，疼痛强烈，甚至影响食欲、睡眠			

③肌肉/关节疼痛

我最近 3 天内有出现不明原因的肌肉/关节疼痛

次数：约（ ）次 或 基本每天都有≥1 次（ ）

严重程度：

0	1	2	3	4	5	6	7	8	9	10
无	有，轻微且尚能忍受			有一定疼痛，会影响睡眠但尚能忍受			有，疼痛强烈，甚至影响食欲、睡眠			

疼痛部位：

颈部	肩部	背部	胸部	胳臂	手	腰部	腹部	臀部	腿部	脚

疼痛性质：

胀痛	酸痛	刺痛	切割痛	绞痛	钝痛	牵扯痛	不易分类

④疲乏/乏力

我最近 3 天内有出现不明原因的疲乏/乏力的感觉

次数：约（ ）次 或 每天断断续续发生（ ）

严重程度：

0	1	2	3	4	5	6	7	8	9	10
无	轻微，但平时并不在意			对日常工作、生活有一定影响，但尚能忍受			对日常工作、生活影响严重			

⑤恶心、反胃

我最近 3 天内有出现不明原因的恶心、反胃感

次数：约（ ）次 或 基本每天都有≥1 次（ ）

严重程度：

0	1	2	3	4	5	6	7	8	9	10
无	轻微，平时并不在意			对日常生活有一定影响，但尚能忍受			对日常生活影响严重			

⑥干呕

我最近 3 天内有出现不明原因的干呕

次数：约（ ）次 或 基本每天都有≥1 次（ ）

严重程度：

0	1	2	3	4	5	6	7	8	9	10
无	轻微，平时并不在意			对日常生活有一定影响，但尚能忍受			对日常生活影响严重			

⑦呕吐（呕吐出呕吐物）

我最近 3 天内有出现不明原因的呕吐（呕吐出呕吐物）

次数：约（ ）次 或 基本每天都有≥1 次（ ）
严重程度：

0	1	2	3	4	5	6	7	8	9	10
无	轻微，平时并不在意			对日常生活有一定影响，但尚能忍受			对日常生活影响严重			

⑧腹泻
我最近 3 天内有出现不明原因的腹泻
次数：约（ ）次 或 基本每天都有≥1 次（ ）
严重程度：

0	1	2	3	4	5	6	7	8	9	10
无	轻微，平时并不在意			对日常生活有一定影响，但尚能忍受			对日常生活影响严重			

13.1.2 第一、第二针疫苗注射后的随访指标

①疫苗注射部位疼痛
在第一/第二针疫苗注射后 3 天内，我有出现疫苗注射部位疼痛，并且我不能否定同疫苗注射后可能的不良反应有关
次数：约（ ）次 或 每天断断续续发生（ ）
严重程度：

0	1	2	3	4	5	6	7	8	9	10
无	有，轻微且尚能忍受			有一定疼痛，会影响工作、生活但尚能忍受			有，疼痛强烈，甚至影响食欲、睡眠			

②头痛
我第一/第二针疫苗注射后 3 天内，有出现头痛，并且我不能否定同疫苗注射后可能的不良反应有关
次数：约（ ）次 或 每天断断续续发生（ ）
严重程度：

0	1	2	3	4	5	6	7	8	9	10
无	有，轻微且尚能忍受			有一定疼痛，会影响睡眠但尚能忍受			有，疼痛强烈，甚至影响食欲、睡眠			

③肌肉/关节疼痛
我第一/第二针疫苗注射后 3 天内，有出现肌肉/关节疼痛，并且我不能否定同疫苗注射后可能的不良反应有关
次数：约（ ）次 或 基本每天都有≥1 次（ ）
严重程度：

0	1	2	3	4	5	6	7	8	9	10

无	轻微，平时并不在意	对日常生活有一定影响，但尚能忍受	对日常生活影响严重

疼痛部位：

颈部	肩部	背部	胸部	胳臂	手	腰部	腹部	臀部	腿部	脚

疼痛性质：

胀痛	酸痛	刺痛	切割痛	绞痛	钝痛	牵扯痛	不易分类

④疲乏/乏力

我第一/第二针疫苗注射后 3 天内，有疲乏/乏力的感觉，并且我不能否定同疫苗注射后可能的不良反应有关

次数：约（　　　）次 或 每天断断续续发生（　　　）

严重程度：

0	1	2	3	4	5	6	7	8	9	10
无	轻微，但平时并不在意			对日常工作、生活有一定影响，但尚能忍受			对日常工作、生活影响严重			

⑤恶心、反胃

我第一/第二针疫苗注射后 3 天内，有出现不明原因的恶心、反胃，并且我不能否定同疫苗注射后可能的不良反应有关

次数：约（　　　）次　或 基本每天都有≥1 次（　　　）

严重程度：

0	1	2	3	4	5	6	7	8	9	10
无	轻微，平时并不在意			对日常生活有一定影响，但尚能忍受			对日常生活影响严重			

⑥干呕

我第一/第二针疫苗注射后 3 天内，有出现不明原因的干呕，并且我不能否定同疫苗注射后可能的不良反应有关

次数：约（　　　）次　或 基本每天都有≥1 次（　　　）

严重程度：

0	1	2	3	4	5	6	7	8	9	10
无	轻微，平时并不在意			对日常生活有一定影响，但尚能忍受			对日常生活影响严重			

⑦呕吐（呕吐出呕吐物）

我第一/第二针疫苗注射后 3 天内，有出现呕吐（呕吐出呕吐物），并且我不能否定同疫苗注射后可能的不良反应有关

次数：约（　　　）次　或 基本每天都有≥1 次（　　　）

严重程度：

0	1	2	3	4	5	6	7	8	9	10
无	轻微，平时并不在意			对日常生活有一定影响，但尚能忍受			对日常生活影响严重			

⑧腹泻
我第一/第二针疫苗注射后 3 天内，有出现腹泻，并且我不能否定同疫苗注射后可能的不良反应有关
次数：约（　　）次 或 基本每天都有≥1 次（　　）
严重程度：

0	1	2	3	4	5	6	7	8	9	10
无	轻微，平时并不在意			对日常生活有一定影响，但尚能忍受			对日常生活影响严重			

13.2 次要指标

次要指标的测量时间为第一、第二针疫苗注射后，其测量方式皆为 VAS 数字模拟评分量表，具体如下：
①我对新冠疫苗注射后可能产生的不良反应之了解程度

0	1	2	3	4
完全不了解	仅仅听说可能会有	一般，有大概了解	相对比较了解	非常清楚

②我对新冠疫苗注射后可能产生的不良反应之担忧程度

0	1	2	3	4
完全不担忧	仅仅极偶尔想到	一般，有一些担忧	相对比较担忧	非常担忧

13.3 安全性指标

安全性指标的测量时间为第一、第二针疫苗注射后立即及分别于注射第一、第二针疫苗后第 3、7、15 天，旨在观察可能的严重不良事件与干预导致的不良反应。介于本研究即为观察研究中医耳穴贴丸是否能够减轻、减少新型冠状病毒疫苗注射后疼痛性（包括注射部位疼痛、头痛、其它肌肉关节疼痛）、乏力性、及胃肠道性（包括恶心呕吐、腹泻）不良反应以及其所能减轻的程度，故无法对中医耳穴贴丸组、假耳穴贴丸组未收到干预下的非严重不良反应进行观察，而空白对照组的疼痛性（包括注射部位疼痛、头痛、其它肌肉关节疼痛）、乏力性、及胃肠道性（包括恶心呕吐、腹泻）不良反应即本研究的主要观

察指标。具体如下：

13.3.1 严重不良事件

严重不良事件报告表（SAE）

临床试验批准文号：　　　　　　　　　　　　　　　　　　　　　　编号：

报告类型	☐首次报告　☐随访报告　☐总结报告　报告时间：　年　月　日
医疗机构及专业名称	电话：
申报单位名称	电话：
试验干预措施名称	中文名称：<br>英文名称：
参试者情况	姓名：　性别：　出生年月：　民族：<br>疾病诊断：
SAE 情况	☐导致住院　☐延长住院时间　☐伤残　☐功能障碍<br>☐导致先天畸形　☐危及生命或死亡　☐其他
SAE 发生时间 ：　年　月　日	SAE 反应严重程度：　☐轻度　☐中度　☐重度
对试验干预措施采取的措施	☐继续使用　☐减少刺激量　☐暂停干预后又恢复<br>☐停用干预措施
SAE 转归	☐症状消失（后遗征：☐有　☐无）　☐症状持续　☐死亡（死亡时间：　年　月　日）
SAE 与试验干预措施的关系	☐肯定有关　☐可能有关　☐可能无关　☐无关　☐无法判定
破盲情况	☐未破盲　☐已破盲（破盲时间：　年　月　日）
SAE 报道情况	国内：　☐有　☐无　☐不详　国外：☐有　☐无　☐不详
SAE 发生及处理的详细情况：	

报告单位名称：　　　　　　　　报告人职务/职称：　　　　　　报告人签名：

13.3.2 不良反应

①在第一/第二针疫苗注射后 3 天内，我有出现贴丸处皮肤过敏等不适

是（　　　）　　　　　　　否（　　　）

严重程度：

0	1	2	3	4	5	6	7	8	9	10
无	偶尔且轻微，平时并不在意			对日常生活有一定影响，但尚能忍受			对日常生活影响严重			

②在第一/第二针疫苗注射后 3 天内，我有出现贴丸处异常疼痛

是（　　　）　　　　　　　否（　　　）

严重程度：

0	1	2	3	4	5	6	7	8	9	10
无	偶尔且轻微，平时并不在意			对日常生活有一定影响，但尚能忍受			对日常生活影响严重			

③在第一/第二针疫苗注射后 3 天内，我有出现贴丸处其它异常

（　　　　　　　　　　　　　　　　　　　　　　　）

是（　　　）　　　　　　　否（　　　）

严重程度：

0	1	2	3	4	5	6	7	8	9	10
无	偶尔且轻微，平时并不在意			对日常生活有一定影响，但尚能忍受			对日常生活影响严重			

十四、对参试者有效性认定的定义

①参试者有效性的定义

签署了知情同意书、筛选合格进入研究、良好依从、按方案及流程要求完成了干预及随访的参试者。

②参试者退出的定义

签署了知情同意书、筛选合格进入研究后在干预前、干预中、干预后随访过程中要求退出的参试者。

③参试者剔除的定义

签署了知情同意书、进入研究但在项目监察中或研究结束后发现不符合纳入标准、属于排除的参试者；

没有签署知情同意书的参试者；

没有按照治疗标准执行干预；依从性差；

未接受干预或不遵守或承担知情协议相应责任与义务；

违背研究方案，没有按照标准执行耳穴贴丸后按摩的参试者；

进入研究后，因各种原因放弃耳穴贴丸或中途不满 5 天自行取下耳穴贴丸的参试者。

④参试者失访的定义

在项目结束前经多次、多方面无法联系到的干预参试者；

没有完成方案所规定观察周期的研究对象，称为脱落，对因不良反应而脱落者，经随访最后判断与试验药物有关者，必须记录在 CRF 中，并通知项目负责人。

对于任何脱落病例，研究者必须在 CRF 表中填写脱落的原因，一般情况下有 6 种，即不良事件、缺乏疗效、违背试验方案（包括依从性差）、失访（包括研究对象自行退出）、项目负责人中止和其他。

对于任何脱落病例完成所能完成的评估项目，并填写研究结论表，尽可能记录最后一次干预及其结局。

⑤参试者混杂的定义

对新冠疫苗注射或临床研究项目过度焦虑、压力过大者。

⑥参试者中止的定义：

项目负责人或研究者综合多方面的因素中止参试者继续研究的参试者；

研究中发现参试者对干预措施过敏者；

取消第二针疫苗注射者。

⑦参试者暂停的定义：

填写了知情同意书后的参试者对本项目持较多疑虑者；

填写了知情同意书后的参试看有意退出对本项目者。

十五、不良事件和不良反应的定义、鉴定方法和管理制度

15.1 不良事件

①定义：是参试者接受干预措施后所发生的任何不良医疗事件，该事件可能与治疗无关。

②不良事件的观察记录：对参试者在试验过程中发生的任何不良事件都必

需仔细询问和追查。所有不良事件都必须判定其性质、严重程度和与干预措施的相关性，并严格记录在病例报告表中。

③相关性评估：不良事件与试验干预措施相关性有：肯定有关,很可能有关,可能有关,可能无关,肯定无关。其中用肯定有关＋很可能有关＋可能有关统计不良反应发生率。

④严重程度评价

不良事件的强度或严重程度按下标准分为三级：

轻度：通常为一过性的，且不影响正常日常活动

中度：相当不适而影响到正常日常活动，参试者自觉症状明显，但可忍受，无须停止干预措施

重度：无法进行正常日常活动，参试者自觉症状显著，不能忍受，需要停止干预措施

⑤试验期间及试验之后对不良事件的处理

研究者应随访观察和记录所有不良事件的转归，跟踪由于不良事件而退出试验的参试者直至不良事件完全解除。研究者必须判断不良事件是否与研究干预措施有关，并提供支持这一判断的依据。

15.2 严重不良事件

①定义：临床试验过程中发生需住院治疗、延长住院时间、伤残、影响工作能力、危及生命或死亡、导致先天畸形等事件。

②严重不良事件的报告

出现严重不良事件时应在 24 小时内报告组长单位及申报单位，并填写严重不良事件表。

严重不良事件报告表（SAE）

临床试验批准文号：　　　　　　　　　　　　　　　　　　　　　　　　　　编号：

报告类型	☐首次报告　☐随访报告　☐总结报告		报告时间：　年　月　日	
医疗机构及专业名称			电话	
申报单位名称			电话	
试验干预措施名称	中文名称：			
	英文名称：			
参试者情况	姓名：	性别：	出生年月：	民族：
	疾病诊断：			
SAE 情况	☐导致住院　☐延长住院时间　☐伤残　☐功能障碍<br>☐导致先天畸形　☐危及生命或死亡　☐其他			
SAE 发生时间 ：　年　月　日	SAE 反应严重程度：　☐轻度　☐中度　☐重度			
对试验干预措施采取的措施	☐继续使用　☐减少刺激量　☐暂停干预后又恢复<br>☐停用干预措施			
SAE 转归	☐症状消失（后遗征：☐有　☐无）　☐症状持续　☐死亡（死亡时间：　年　月　日）			
SAE 与试验干预措施的关系	☐肯定有关　☐可能有关　☐可能无关　☐无关　☐无法判定			
破盲情况	☐未破盲　☐已破盲（破盲时间：　年　月　日）			
SAE 报道情况	国内：☐有　☐无　☐不详　　国外：☐有　☐无　☐不详			
SAE 发生及处理的详细情况：				

报告单位名称：　　　　　　　　　　报告人职务/职称：　　　　　　　　报告人签名：

15.3 不良反应

耳穴贴丸不良反应少见，可能的不良反应包括局部过敏、异常疼痛、炎症。若出现不良反应，立即停止干预，轻轻撕下耳穴帖，行局部一般常规处理。

①在第一/第二针疫苗注射后 3 天内，我有出现贴丸处皮肤过敏等不适

是（ ） 否（ ）

严重程度：

<table>
<tr><td>0</td><td>1</td><td>2</td><td>3</td><td>4</td><td>5</td><td>6</td><td>7</td><td>8</td><td>9</td><td>10</td></tr>
<tr><td>无</td><td colspan="3">偶尔且轻微，平时并不在意</td><td colspan="3">对日常生活有一定影响，但尚能忍受</td><td colspan="4">对日常生活影响严重</td></tr>
</table>

②在第一/第二针疫苗注射后 3 天内，我有出现贴丸处异常疼痛

是（ ） 否（ ）

严重程度：

<table>
<tr><td>0</td><td>1</td><td>2</td><td>3</td><td>4</td><td>5</td><td>6</td><td>7</td><td>8</td><td>9</td><td>10</td></tr>
<tr><td>无</td><td colspan="3">偶尔且轻微，平时并不在意</td><td colspan="3">对日常生活有一定影响，但尚能忍受</td><td colspan="4">对日常生活影响严重</td></tr>
</table>

③在第一/第二针疫苗注射后 3 天内，我有出现贴丸处其它异常

（ ）

是（ ） 否（ ）

严重程度：

<table>
<tr><td>0</td><td>1</td><td>2</td><td>3</td><td>4</td><td>5</td><td>6</td><td>7</td><td>8</td><td>9</td><td>10</td></tr>
<tr><td>无</td><td colspan="3">偶尔且轻微，平时并不在意</td><td colspan="3">对日常生活有一定影响，但尚能忍受</td><td colspan="4">对日常生活影响严重</td></tr>
</table>

15.4 试验中紧急破盲方法

若参试者出现严重的不良反应，必要时可由中心负责人开启应急信封紧急破盲，并在病例观察表中记录打开的原因、日期。随后通知组长单位和申办单位。已被解盲的参试者需要研究者与申办者讨论决定该参试者是否从研究中退出。

十六、伦理考量

16.1 伦理委员会的选择

成都中医药大学附属医院医学伦理委员会人员组成符合我国法规要求，委员包含医学、药学、护理学、法律学等专业，以及社区代表。所有委员均经过 GCP 及医学伦理相关培训。本伦理委员会依据并严格遵守伦理审查相关法规开展工作，主要受理范围包括药物、医疗器械、临床科研项目的伦理审查，审查类别包括初始审查、复审、年度/定期跟踪审查、修正案审查、严重不良事件审查、违背方案审查、暂停/终止研究审查、研究完成审查等。

16.2 报批程序

①申请人携带课题立项证明材料到伦理委员会办公室进行项目信息的登记，办理伦理审查费用缴纳事务，获取伦理审查平台系统的用户名和初始密码，同时指定该项目伦理审查的项目管理秘书；

②申请人与管理秘书联系，或登录官网，下载《伦理审查申请指南》，按照附件中《送审文件清单》的要求准备电子版材料；并通过“医院官网-科学研究-伦理审查-伦理审查系统”中在线提交伦理审查材料；

③项目管理秘书接受材料后，进行形式审查，在 5 个工作日内发放《补充/修改送审材料通知》，如此往复，直至形式审查合格，发放《伦理审查受理通知》；

④形式审查合格后，申请人请准备相应份数的纸质版材料，提交至伦理办公室；

⑤按照伦理委员会标准操作规程（SOP），多数项目的初始审查方式为会议审查，少数为快速审查。秘书在收到全部材料,确定受理后，在 5 个工作日内，联系主审委员进行审查。若快速审查意见为“作必要修正后重审、不同意、终止或暂停已批准的研究、提交会议审查”，则转为会议审查的方式；

⑥如初始审查意见为修正后同意或修正后重审，伦理委员会将以伦理审查意见的形式告知申请人；申请人修改方案后，提交复审申请，经主审委员再次审查通过后，领取伦理审查批件，开始临床研究；

⑦申请人按照伦理委员会审查通过的方案开始临床研究，研究过程中如需对研究方案进行改动、或发生违背方案、可疑且非预期的严重不良反应（SUSAR）、或需提前终止/暂停临床研究等，则须按指南要求递交相应表格及文件；项目启动后，应按照规定的跟踪审查频率及时递交《研究进展报告》；

⑧申请人完成临床试验，应及时向伦理委员会提交研究完成报告（需提供临床试验结果的摘要）。

16.3 知情同意过程

每一位参试者纳入本研究前，研究者须给每位患者一份书面知情同意书，由参与本研究的参试者的纳入研究者以书面文字形式，向参试者或其指定代表完整、全面地介绍本研究的目的、程序和可能的风险和获益，且让患者知道他们有权随时退出本研究。

研究医师有责任在每位参试者进入研究之前让参试者充分知情并由参试者签署知情同意书确认，知情同意书将作为临床研究文挡保留备查。

16.4 注册时间与注册机构

①本研究将在正式纳入参试者前在中国临床研究中心完成注册；

②本研究已经于 2021 年 02 月 04 日通过成都中医药大学伦理委员会正式会议伦理审查，审查通过批件号为：2021KL-015

十七、参试者的征募

①参试者征募地点：

成都中医药大学附属医院；

成都市金牛区西安路社区卫生服务中心；

成都市武侯区望江路社区卫生服务中心；

成都市武侯区浆洗街社区卫生服务中心；

成都市青羊区太升社区卫生服务中心；

成都市青羊区草堂社区服务中心。

②征募方法：制作研究项目参试者招募海报、微信等招募

③筛选过程：

首先将符合新冠疫苗注射条件且愿意按照相关要求完成两针疫苗注射者为初步筛选对象；

再以平时不存在慢性/习惯性性/持续性疫苗注射部位疼痛、疲乏/乏力、腹泻、头痛、疼痛、恶心/反胃、干呕、呕吐（呕吐出呕吐物），及未患有相关疾病为标准筛选参试者；

再按照纳入、排除标准进行严格的筛选；

最后按照是否决定自愿参加本研究和签署知情同意书筛选。

④实施筛选的研究人员：由本研究负责研究数据收集的研究人员独立进行。

⑤监察员：对实施筛选的病例进行监察和质量控制，由本研究研究质量控制人员监察。

十八、参试者一般信息的收集

参试者一般信息的收集由本研究中负责研究数据收集的研究人员独立进行。需收集的参试者一般信息主要包括：

姓名、性别、电话、年龄、文化水平、职业、吸烟情况及饮酒情况。

在参试者一般信息的收集过程中将注意其信息的保密，专人专管。

十九、基线指标和观测项目

19.1 第一、第二针疫苗注射前的基线指标

①疫苗注射部位疼痛

在疫苗注射前，我最近 3 天内有出现不明原因的疫苗注射部位疼痛

次数：约（　　　）次 或 每天断断续续发生（　　　）

严重程度：

0	1	2	3	4	5	6	7	8	9	10
无	有，轻微且尚能忍受			有一定疼痛，会影响工作、生活但尚能忍受			有，疼痛强烈，甚至影响食欲、睡眠			

②头痛

我最近 3 天内有出现不明原因的头痛

次数：约（　　）次 或 每天断断续续发生（　　）

严重程度：

0	1	2	3	4	5	6	7	8	9	10
无	有，轻微且尚能忍受			有一定疼痛，会影响睡眠但尚能忍受			有，疼痛强烈，甚至影响食欲、睡眠			

③肌肉/关节疼痛

我最近 3 天内有出现不明原因的肌肉/关节疼痛

次数：约（　　）次 或 基本每天都有≥1 次（　　）

严重程度：

0	1	2	3	4	5	6	7	8	9	10
无	有，轻微且尚能忍受			有一定疼痛，会影响睡眠但尚能忍受			有，疼痛强烈，甚至影响食欲、睡眠			

疼痛部位：

颈部	肩部	背部	胸部	胳臂	手	腰部	腹部	臀部	腿部	脚

疼痛性质：

胀痛	酸痛	刺痛	切割痛	绞痛	钝痛	牵扯痛	不易分类

④疲乏/乏力

我最近 3 天内有出现不明原因的疲乏/乏力的感觉

次数：约（　　）次 或 每天断断续续发生（　　）

严重程度：

0	1	2	3	4	5	6	7	8	9	10
无	轻微，但平时并不在意			对日常工作、生活有一定影响，但尚能忍受			对日常工作、生活影响严重			

⑤恶心、反胃

我最近 3 天内有出现不明原因的恶心、反胃感

次数：约（　　）次 或 基本每天都有≥1 次（　　）

严重程度：

0	1	2	3	4	5	6	7	8	9	10
无	轻微，平时并不在意			对日常生活有一定影响，但尚能忍受			对日常生活影响严重			

⑥干呕

我最近 3 天内有出现不明原因的干呕

次数：约（ ）次 或 基本每天都有≥1 次（ ）
严重程度：

0	1	2	3	4	5	6	7	8	9	10
无	轻微，平时并不在意			对日常生活有一定影响，但尚能忍受			对日常生活影响严重			

⑦呕吐（呕吐出呕吐物）
我最近 3 天内有出现不明原因的呕吐（呕吐出呕吐物）
次数：约（ ）次 或 基本每天都有≥1 次（ ）
严重程度：

0	1	2	3	4	5	6	7	8	9	10
无	轻微，平时并不在意			对日常生活有一定影响，但尚能忍受			对日常生活影响严重			

⑧腹泻
我最近 3 天内有出现不明原因的腹泻
次数：约（ ）次 或 基本每天都有≥1 次（ ）
严重程度：

0	1	2	3	4	5	6	7	8	9	10
无	轻微，平时并不在意			对日常生活有一定影响，但尚能忍受			对日常生活影响严重			

19.2 第一、第二针疫苗注射后的观测项目

①疫苗注射部位疼痛
在第一/第二针疫苗注射后 3 天内，我有出现疫苗注射部位疼痛，并且我不能否定同疫苗注射后可能的不良反应有关
次数：约（ ）次 或 每天断断续续发生（ ）
严重程度：

0	1	2	3	4	5	6	7	8	9	10
无	有，轻微且尚能忍受			有一定疼痛，会影响工作、生活但尚能忍受			有，疼痛强烈，甚至影响食欲、睡眠			

②头痛
我第一/第二针疫苗注射后 3 天内，有出现头痛，并且我不能否定同疫苗注射后可能的不良反应有关
次数：约（ ）次 或 每天断断续续发生（ ）
严重程度：

0	1	2	3	4	5	6	7	8	9	10

无	有，轻微且尚能忍受	有一定疼痛，会影响睡眠但尚能忍受	有，疼痛强烈，甚至影响食欲、睡眠

③肌肉/关节疼痛

我第一/第二针疫苗注射后 3 天内，有出现肌肉/关节疼痛，并且我不能否定同疫苗注射后可能的不良反应有关

次数：约（　　）次 或 基本每天都有≥1 次（　　）

严重程度：

0	1	2	3	4	5	6	7	8	9	10
无	轻微，平时并不在意			对日常生活有一定影响，但尚能忍受			对日常生活影响严重			

疼痛部位：

颈部	肩部	背部	胸部	胳臂	手	腰部	腹部	臀部	腿部	脚

疼痛性质：

胀痛	酸痛	刺痛	切割痛	绞痛	钝痛	牵扯痛	不易分类

④疲乏/乏力

我第一/第二针疫苗注射后 3 天内，有疲乏/乏力的感觉，并且我不能否定同疫苗注射后可能的不良反应有关

次数：约（　　）次 或 每天断断续续发生（　　）

严重程度：

0	1	2	3	4	5	6	7	8	9	10
无	轻微，但平时并不在意			对日常工作、生活有一定影响，但尚能忍受			对日常工作、生活影响严重			

⑤恶心、反胃

我第一/第二针疫苗注射后 3 天内，有出现不明原因的恶心、反胃，并且我不能否定同疫苗注射后可能的不良反应有关

次数：约（　　）次　或 基本每天都有≥1 次（　　）

严重程度：

0	1	2	3	4	5	6	7	8	9	10
无	轻微，平时并不在意			对日常生活有一定影响，但尚能忍受			对日常生活影响严重			

⑥干呕

我第一/第二针疫苗注射后 3 天内，有出现不明原因的干呕，并且我不能否定同疫苗注射后可能的不良反应有关

次数：约（　　）次　或 基本每天都有≥1 次（　　）

严重程度：

0	1	2	3	4	5	6	7	8	9	10
无	轻微，平时并不在意			对日常生活有一定影响，但尚能忍受			对日常生活影响严重			

⑦呕吐（呕吐出呕吐物）
我第一/第二针疫苗注射后 3 天内，有出现呕吐（呕吐出呕吐物），并且我不能否定同疫苗注射后可能的不良反应有关
次数：约（ ）次 或 基本每天都有≥1 次（ ）
严重程度：

0	1	2	3	4	5	6	7	8	9	10
无	轻微，平时并不在意			对日常生活有一定影响，但尚能忍受			对日常生活影响严重			

⑧腹泻
我第一/第二针疫苗注射后 3 天内，有出现腹泻，并且我不能否定同疫苗注射后可能的不良反应有关
次数：约（ ）次 或 基本每天都有≥1 次（ ）
严重程度：

0	1	2	3	4	5	6	7	8	9	10
无	轻微，平时并不在意			对日常生活有一定影响，但尚能忍受			对日常生活影响严重			

19.3 次要指标

次要指标的测量时间为第一、第二针疫苗注射后，其测量方式皆为 VAS 数字模拟评分量表，具体如下：

①我对新冠疫苗注射后可能产生的不良反应之了解程度

0	1	2	3	4
完全不了解	仅仅听说可能会有	一般，有大概了解	相对比较了解	非常清楚

②我对新冠疫苗注射后可能产生的不良反应之担忧程度

0	1	2	3	4
完全不担忧	仅仅极偶尔想到	一般，有一些担忧	相对比较担忧	非常担忧

19.4 严重不良事件

严重不良事件报告表（SAE）

临床试验批准文号：

编号：

<table>
<tr><td>报告类型</td><td colspan="4">☐首次报告　　　☐随访报告　　　☐总结报告</td><td colspan="2">报告时间：　年　月　日</td></tr>
<tr><td>医疗机构及专业名称</td><td colspan="4"></td><td>电话</td><td></td></tr>
<tr><td>申报单位名称</td><td colspan="4"></td><td>电话</td><td></td></tr>
<tr><td rowspan="2">试验干预措施名称</td><td colspan="6">中文名称：</td></tr>
<tr><td colspan="6">英文名称：</td></tr>
<tr><td rowspan="2">参试者情况</td><td>姓名：</td><td>性别：</td><td colspan="2">出生年月：</td><td colspan="2">民族：</td></tr>
<tr><td colspan="6">疾病诊断：</td></tr>
<tr><td>SAE 情况</td><td colspan="6">☐导致住院　☐延长住院时间　☐伤残　☐功能障碍
☐导致先天畸形　☐危及生命或死亡　☐其他</td></tr>
<tr><td colspan="2">SAE 发生时间 ：　年　月　日</td><td colspan="5">SAE 反应严重程度：　☐轻度　☐中度　☐重度</td></tr>
<tr><td>对试验干预措施采取的措施</td><td colspan="6">☐继续使用　☐减少刺激量　☐暂停干预后又恢复
☐停用干预措施</td></tr>
<tr><td>SAE 转归</td><td colspan="6">☐症状消失（后遗征：☐有　☐无）　☐症状持续　☐死亡（死亡时间：　年　月　日）</td></tr>
<tr><td>SAE 与试验干预措施的关系</td><td colspan="6">☐肯定有关　☐可能有关　☐可能无关　☐无关　☐无法判定</td></tr>
<tr><td>破盲情况</td><td colspan="6">☐未破盲　☐已破盲（破盲时间：　年　月　日）</td></tr>
<tr><td>SAE 报道情况</td><td colspan="6">国内：☐有　☐无　☐不详　　　国外：☐有　☐无　☐不详</td></tr>
<tr><td colspan="7">SAE 发生及处理的详细情况：</td></tr>
</table>

报告单位名称：　　　　　　报告人职务/职称：　　　　　　报告人签名：

19.5 不良反应

①在第一/第二针疫苗注射后 3 天内，我有出现贴丸处皮肤过敏等不适
是（　　　）　　　　　　　　否（　　　）
严重程度：

0	1	2	3	4	5	6	7	8	9	10
无	偶尔且轻微，平时并不在意			对日常生活有一定影响，但尚能忍受			对日常生活影响严重			

②在第一/第二针疫苗注射后 3 天内，我有出现贴丸处异常疼痛
是（　　　）　　　　　　　　否（　　　）
严重程度：

0	1	2	3	4	5	6	7	8	9	10
无	偶尔且轻微，平时并不在意			对日常生活有一定影响，但尚能忍受			对日常生活影响严重			

③在第一/第二针疫苗注射后 3 天内，我有出现贴丸处其它异常
（　　　　　　　　　　　　　　　　　　　　　　　　）
是（　　　）　　　　　　　　否（　　　）
严重程度：

0	1	2	3	4	5	6	7	8	9	10
无	偶尔且轻微，平时并不在意			对日常生活有一定影响，但尚能忍受			对日常生活影响严重			

二十、标准操作规程

20.1 中医耳穴贴丸

耳穴贴丸组与假耳穴贴丸组均使用“子彧”牌耳穴贴丸，其中间贴有小的王不留行籽。王不留行籽为黑色、圆形、极小且无特殊气味物。由河北贺氏医疗器械有限公司生产，批号：Q/HHS 01-2016，有效期：3 年。

20.2 耳穴选择

①耳穴贴丸组：在临床试验开始之前，将依据国际标准耳穴指南、专家共识意见与前期临床研究发现的可能的新冠疫苗注射后常见不良反应选取的耳部穴位标记于耳穴简笔图上。耳穴贴丸组所选取耳穴包括（每次皆双侧贴丸）：

神门（TF4）、脾（CO13）、心（CO15）、皮质下（AT4）、交感（AH6a）

②假耳穴贴丸组：在临床试验开始之前，将依据国际标准耳穴指南、专家咨询意见与前期临床研究发现的可能的新冠疫苗注射后常见不良反应选取与新型冠状病毒疫苗注射后常见不良反应无关的耳部区域标记于耳穴简笔图上。假耳穴贴丸组所选取耳穴包括（每次皆双侧贴丸）：

肛门（HX5）、尿道（HX3）、轮 1（HX9）、轮 2（HX10）、轮 3（HX11）

③空白对照组：新冠疫苗注射后不进行特殊干预，与前两组同时随访观察。

20.3 耳穴贴丸与按压操作方法

研究人员依据已经标记好的耳穴简笔图进行耳穴贴丸。首先使用消毒棉签蘸取 75%医用消毒酒精擦拭参试者耳部拟贴丸处，并使用干消毒棉签蘸干。然后使用医用镊子夹取耳穴贴丸轻轻贴于耳穴上，并稍用力按压。

耳穴贴丸组与假耳穴贴丸组共贴丸两次，第一次为新冠疫苗注射后立即耳穴贴丸，第二次为第二针新冠疫苗注射后再次立即双侧耳穴贴丸。贴完后嘱参试者每日自行耳部按压 3-4 次，每次 1 分钟左右。每次耳穴贴丸贴好后保留时间为 5 天。空白对照组则在新冠疫苗注射后不进行特殊干预，与前两组同时随访观察。

20.4 其它注意事项

①参试者在接受耳穴贴丸后，注意嘱其对称轻轻用力按压贴丸处，但不可揉搓或用力过大；注意防止胶布潮湿或污染，以免引发皮肤炎症；

②若出现贴丸处严重疼痛或局部胶布过敏，应立即轻轻撕下耳贴，及时上报，注意观察，酌情处理。

二十一、统计分析方法

数据将使用 SPSS 软件 V.25.0 进行分析。完成试验的参试者将采用按方案（PP）分析，最初纳入随机分组的参试者将采用意向治疗分析（ITT）。

三组基线差异比较采用单因素方差分析（定量资料）和 x 2 检验（分类资料）。有效性评估包括组间和组内比较。采用单因素方差分析和最小显著性差异事后检验比较三组干预前后结果得分的变化。采用配对样本 t 检验比较干预前后平均结果得分的组内方差。

所有概率都是双侧的，显著性水平 $p<0.05$。

二十二、参试者管理制度

本研究参试者为完成新冠疫苗接种者，对其进行入组前、干预前、干预后及随访宣教和管理。

22.1 对新冠疫苗接种者进行研究前的宣教

①宣教人员：本研究人员轮流进行；

②宣教内容：交代介绍本项目＋知情同意书内容，主要包括以下方面

目的：研究中医耳穴贴丸是否能够减轻、减少新型冠状病毒疫苗注射后疼痛性（包括注射部位疼痛、头痛、其它肌肉关节疼痛）、乏力性、及胃肠道性（包括恶心呕吐、腹泻）不良反应以及其所能减轻的程度；

具体干预：双耳中医耳穴贴丸/双耳假耳穴贴丸/空白对照；

干预时间：共贴丸 2 次，耳穴贴丸刺激共 10 天，随访共 6 次。共为期约 30 天（具体取决于两针疫苗接种中隔时间）；

分组情况的交代：每人分到 3 组的机会相同，分组按照参试者进入研究的编号顺序曲随机数字表决定；

观察随访期限：注射第一、第二针疫苗时及分别于注射第一、第二针疫苗后第 3、7、15 天，共为期约 30 天（具体取决于两针疫苗接种中隔时间）；

患者的获益和风险：中医耳穴贴丸组给予双侧对症中医耳穴贴丸，假耳穴贴丸组给予双侧假耳穴贴丸，空白对照组予以空白对照干预；中医耳穴贴丸不良反应极少，可能的不良反应包括局部疼痛、贴丸处胶布过敏；

③宣教方式：口头宜教及纸质宣教说明；

④直教时间：分别于第一/第二次耳穴贴丸后立即进行。

22.2 对纳入参试者干预的管理

①对参试者宣教内容：

1）参试者应如实向研究者说明既往史、现病史、传染病史、药物过敏史等个人情况；

2）参试者应如实向研究者说明平时是否存在慢性/习惯性性/持续性疫苗注射部位疼痛、疲乏/乏力、腹泻、头痛、疼痛、恶心/反胃、干呕、呕吐（呕吐出呕吐物），及是否患有相关疾病；

3）参试者如合并有其他疾病需采取其它检查和治疗，应及时记录，并在随访时说明；

4）参试者应按规定参与随访（线上或线下），不得由他人替代；

5）参试者不得随意将研究资料对外泄漏，不得随意翻阅病例，如有异议，及时与研究者联系；

6）参试者如研究过程中身体发生任何不适，应及时向研究者说明，及时进行检查和治疗；

②宣教的时间：耳穴贴丸（或空白对照）前，对决定参加本项目的参试者；

③直教的人员：本研究人员轮流进行。

④保证依从性的方法：定期电话、微信随诊

二十三、标本管理制度

本研究不涉及任何标本采集、保管、送检。

二十四、药品和器材管理制度

本试验所用中医耳穴贴丸及其它试验用品（包括 75%医用消毒酒精、玻璃酒精瓶、免洗手消、收纳盒、医用镊子、消毒棉签、防护面罩、耳穴贴丸、一次性防护手套、医用外科口罩，其中免洗手消、防护面罩、一次性防护手套、医用外科口罩为为项目操作人员准备的个人防护用品）全部按成都中医药大学附属医院伦理委员会规章管理。各中心接受中医耳穴贴丸及其它试验用品须签

署试验用品接收单，双人签字，一式 2 份，组长单位和各试验中心各执一份。中医耳穴贴丸及其它试验用品置常温、避光、干燥保存，各研究中心需设专人、专柜、上锁保管。每一份中医耳穴贴丸及其它试验用品的发放及回收均应在专门记录单上及时记录。中医耳穴贴丸及其它试验用品管理人员将编有顺序号码的中医耳穴贴丸及其它试验用品按参试者就诊先后顺序依次发给，每个参试者只能使用一个编码。研究结束收回剩余中医耳穴贴丸及其它试验用品，双方签署回收单。

研究中及时收回剩余中医耳穴贴丸及其它试验用品，并妥善保管好所有试验用品和应急信封，研究结束后一并交给申办者。

二十五、数据管理制度

25.1 数据管理的目的

把研究对象的数据迅速、完整、无误地纳入报告，所有涉及数据管理的各种步骤均需记录在案，以便对数据质量及试验实施进行检查。保证数据库的保密性，应具有计算机数据库的维护和支持程序。试验前需设计可被计算机阅读和输入的临床报告表；

研究对象分配必须按试验设计确定的随机方案进行，每名研究对象的密封代码应由研究者保存。

25.2 原始资料记录的制度

1）在研究开始前，项目主持人应与研究人员、监查员讨论如何在原始资料中记录有关临床研究的信息，并建立对原始记录的要求；

2）项目主持人提供原始资料中临床研究信息记录的格式（CRF 表）；

3）原始资料按医疗文件的行业惯例由完成的研究人员签字并注明日期；

4）所有原始资料的更正应由进行更正的研究者签名并注明日期；

5）所有在病例报告表上记录的信息和数据，均应出自原始资料中的记录；

6）病例报告表的填写与移交：CRF 填写是在患者入组后由项目指定专人按照参试者病例号如实填写患者入组信息编号并录入 CRF 表，在填写完成后由参

加纳入患者的研究者审核后签名。

7）数据的录入与修改：在各阶段录入的自参加纳入患者的研究者审核签名、监察员签字后将锁定所录入的该阶段的相关病历信息。所有数据将采用材算机软件编制数据录入程序进行双份录入。在此期间，将有疑问的 CRF 表通过临床监查员转交研究者进行数据审核， 研究者应尽快回答并返回。在盲态审核并认为所建立的数据库正确后，将由主要研究者、统计分析人员和数据管理监督管理人员对数据进行锁定。

8）资料保存：所有与本次临床试验有关的研究资料在研究结束后保存在成都中医药大学附属医院集中保管至少 5 年。

25.3 采集和录入（记录）人员

病例报告表由指定受过培训的固定专人填写，但所有各步骤的数据信息等需纳入参试者的研究者仔细检查、核对签字确认，标明日期。

25.4 核对制度

1）病例报告表填写人对所填写数据的实时核对并签字（填写人、纳入参试者的研究者)；

2）研究监察员不定期监察核实各主要点的数据录入是否正确、准确无误并签字；

3）耳穴贴丸时的核对。

二十六、数据安全与监察委员会的组成和工作职责

25.1 数据安全

1）临床研究数据包括与干预究所用干预措施研究有关的一切数据资料：研究者的情况、研究对象的基本情况、CRF 表的情况、各种相关指标实测值等。

2）应详细、及时、准确登记试验的各种数据。

3）对实施的指标测量应如实填写，并注明原因。

4）研究结束后，总结资料时应如实统计各种数据。

5）对不能完成的指标测量情况应在测量前向组长单位和申办者说明。

6）数据修改处应有修改人的签名并注明时间，必要时说明理由。

25.2 监察委员会的组成和工作职责

监察员：由不同参与单位的主持人或其同单位的一个熟悉相关领域临床、具有研究生学历、曾参与过 RCT 研究或参加过 GCP 相关培训，并具备相应知识的医生担任。监察员在项目开始前需经过必要的培训，了解其职责。

监察委员会的组成：项目主持人＋分中心负责人＋监察员

监察委员会的工作职责：监察员应遵循标准操作规程，督促临床研究的进行，以保证临床研究按方案执行，具体包括：

①在研究前确认承担单位具有适当条件、足够数量的研究对象，参与研究人员已熟悉方案要求；

②在研究过程中监察研究者对研究方案的执行情况，确认在研究前所有研究对象签署了知情同意书，了解研究对象的入选率及试验进展状况，确认入选的研究对象是否合格；

③确认所有数据的记录与报告正确完整，所有病例报告表填写正确，并与原始资料一致， 所有错误或遗漏均已改正或注明，签名并注明日期。每一个研究对象的干预措施改变、治疗变更、合并用药、并发疾病、失访、检查遗漏等均应确认并记录。核实入选研究对象的退出与失访并应在病例报告表中予以说明；

④确认所有不良事件均记录在案，严重不良事件在规定时间内作出报告并记录在案；

⑤核实研究用物资按照有关规定供应、储藏、分发，并做相应的记录；

⑥ 协助研究者进行必要的通知及申请，向项目主持人报告研究数据和结果；

⑦应清楚、如实记录研究者未能做到的随访，未进行的检查、记录，以及是否对错误、遗漏作出纠正；

⑧每次监察后在监察文档中签字，并作一书面报告递送项目主持人，报告应述明监察日期、时间、监察员姓名、监察的发现等。

二十七、研究团队

姓名	性别	职称	学历	专业	是否经过 GCP 培训	研究分工
张勤修	男	二级教授/主任医师	博士	耳鼻咽喉科学	是	研究统筹安排/学术顾问/技术指导/研究质控
蒋路云	男	教授/主任医师	硕士	耳鼻咽喉科学	否	研究学术顾问/数据管理
谢慧	女	教授/主任医师	博士	中医五官科学	否	研究学术顾问/研究质控
周立	男	副教授/副主任医师	博士	耳鼻咽喉科学	否	研究学术顾问
李昕蓉	女	教授/副主任医师	博士	耳鼻咽喉科学	是	研究技术指导/研究质控
刘洋	男	主治医师	博士	中医五官科学	否	研究技术指导
徐万水	男	主任医师	硕士	临床医学	否	分中心研究统筹安排/研究质控
杨文生	男	主任医师	硕士	临床医学	否	分中心研究统筹安排/研究质控
罗红艳	女	主任医师	硕士	临床医学	否	分中心研究统筹安排/研究质控
郭萍	女	主任医师	硕士	临床医学	否	分中心研究统筹安排/研究质控
梁红	女	主任医师	硕士	临床医学	否	分中心研究统筹安排/研究质控
傅勤为	男	无（研究生）	硕士在读	中医五官科学	否	研究具体策划/细节流程安排/报告撰写
冯成郅	男	无（本科生）	本科在读	针灸推拿学	否	研究数据收集
王寻雨	女	无（研究生）	硕士在读	中医五官科学	否	数据录入/研究数据收集/数据管理
赵茂兰	女	无（研究生）	硕士在读	针灸推拿学	否	研究数据收集

吴宣諭	女	无（本科生）	本科在读	中医学	否	研究数据收集
胡思瀚	男	无（本科生）	本科在读	中医学	否	研究数据收集
陈曦	女	无（本科生）	本科在读	中医学	否	研究数据收集
罗明碧	女	无（研究生）	硕士在读	针灸推拿学	否	研究数据收集
王智巧	女	无（研究生）	硕士在读	中医五官科学	否	数据统计
林瀚文	女	无（本科生）	本科在读	中医学	否	数据录入/数据统计
许果	女	无（本科生）	本科在读	针灸推拿学	否	数据统计
萧智勇	男	无（研究生）	硕士在读	针灸推拿学	否	数据录入/数据统计
蔡梦圆	女	无（研究生）	硕士在读	中医内科学	否	数据统计/数据管理
王丽	女	无（本科生）	本科在读	针灸推拿学	否	数据录入/数据统计
敖之敏	女	无（本科生）	本科在读	针灸推拿学	否	干预措施实施/物资管理
蒲涛	女	无（本科生）	本科在读	中医学	否	干预措施实施/物资管理
苏畅	女	无（本科生）	本科在读	临床医学	否	干预措施实施/物资管理
黄佳丽	女	无（本科生）	本科在读	针灸推拿学	否	干预措施实施/物资管理
王朝雨	女	无（本科生）	本科在读	针灸推拿学	否	干预措施实施
唐金凡	男	无（研究生）	硕士在读	针灸推拿学	否	干预措施实施/报告撰写
周静	女	无（研究生）	硕士在读	中医五官科学	否	干预措施实施/报告撰写
肖欢	女	无（研究生）	硕士在读	中医五官科学	否	数据管理

二十八、知识产权

本研究知识产权的归属为项目参与成员，最终解释权归项目负责人所有。作者署名政策遵循国际 ICMJE 规则，论文作者必须是直接参与论文选题、设计、研究、资料分析与解释的全部或部分主要工作,或撰写论文关键内容,能对

论文内容负责者，其作者顺序依据对研究的实际贡献。我们将在中国临床试验注册中心完成注册后的一周内于《Trials》期刊以开放获取（Open Access）途径快速公开发表本研究研究计划，并及时将研究计划链接于本平台（中国临床试验注册中心）更新。

二十九、发表计划

本研究预计在研究随访结束 2 个月内与经同行评审的相关学术期刊发表研究报告≥2 篇。由于随访时间相对较短，故不准备发表阶段性总结报告。

三十、原始数据共享计划

本研究原始数据将在研究随访结束 3 个月内上传至本平台（中国临床试验注册中心）用于公众共享。

三十一、试验结束后对参试者的治疗和管理

本研究为对中医耳穴贴丸缓解新冠病毒疫苗注射后可能的疼痛性、 乏力性及胃肠道性不良反应的疗效评价。如在本研究为期约 30 天的随访过程中仍有参试者存在新冠病毒疫苗注射后可能的疼痛性、 乏力性及胃肠道性不良反应未获改善，参试者（疫苗接种者）需要遵照疫苗注射点相关规定根据具体情况进一步上报，并咨询相关处理措施。

参考文献

1. Zhu FC, Guan XH, Li YH, Huang JY, Jiang T, Hou LH, et al,. Immunogenicity and safety of a recombinant adenovirus type-5-vectored COVID-19 vaccine in healthy adults aged 18 years or older: a randomised, double-blind, placebo-controlled, phase 2 trial. Lancet. 2020 Aug 15;396(10249):479-488.

2. Ramasamy MN, Minassian AM, Ewer KJ, Flaxman AL, Folegatti PM, Owens DR, et al,. Safety and immunogenicity of ChAdOx1 nCoV-19 vaccine administered in a prime-boost regimen in young and old adults (COV002): a single-blind, randomised, controlled, phase 2/3 trial. Lancet. 2021 Dec 19;396(10267):1979-1993.

3. 文婷,李干,陈世彪,刘佳.耳穴磁珠贴压对分娩镇痛中产妇体温、炎性反应及胎盘病理结果的

影响[J].针刺研究,2020,45(12):1010-1013.

4. 谷荣.耳穴贴压在康复科脑卒中恢复期参试者便秘中的应用[J].中西医结合心血管病电子杂志,2020,8(35):153+165.

5. 尹昊,马丹,孟鸿雁.耳穴压丸疗法在高危儿早期干预中的运用[J].中医研究,2020,33(12):24-26.

6. Hou, Pu-Wei et al. "The History, Mechanism, and Clinical Application of Auricular Therapy in Traditional Chinese Medicine." Evidence-based complementary and alternative medicine : eCAM vol. 2015 (2015): 495684.

7. 刘静娜,谢学慧,曹桂娴,梁天山.耳穴贴压法治疗在老年人原发性高血压护理中的应用及对血压水平的影响[J].国际护理学杂志,2020,39(22):4095-4099.

8. 范学宇.耳穴贴压在妇科疾病参试者宫腔镜检查中的镇痛作用[J].医疗装备,2020,33(20):64-65.

9. 杨超,马艳,梅俊华,龚雪,王梦,刘坤.八段锦联合耳穴贴压治疗新型冠状病毒肺炎伴失眠疗效观察[J/OL].中国针灸:1-4[2021-01-06].

10. Zhou, Guangdong et al. "In Vitro Regeneration of Patient-specific Ear-shaped Cartilage and Its First Clinical Application for Auricular Reconstruction." EBioMedicine vol. 28 (2018): 287-302.

11. 薛新丽,杨瑞.耳穴压丸配合针刺治疗阴虚火旺型失眠的疗效观察[J].中医外治杂志,2020,29(05):16-17.

12. 李蕾蕾,刘芝修,李晶晶,刘静,陈桂兰,董婷.耳穴贴压合肝豆灵对肝豆状核变性参试者抑郁状态的影响[J].安徽中医药大学学报,2020,39(05):51-53.

13. 高爱梅,崔芹,赵春丽,刘阳.耳穴压丸配合神经肌肉电刺激对产后排尿困难的影响[J].吉林中医药,2020,40(09):1206-1208.

14. Yin, Chunfang et al. "Clinical application of auricular point sticking in perioperative hemostasis for elderly patients with intertrochanteric fractures of the femur." Medicine vol. 98,35 (2019): e16963.

15. 马丽芳,卿雁冰,岳树锦,苏春香.耳穴贴压改善围绝经期女性失眠的系统综述[J].中华现代护理杂志,2020,26(26):3620-3625.

16. 刘书齐.耳穴贴压法对肺癌术后参试者睡眠质量的影响研究[J].世界睡眠医学杂志,2020,7(08):1327-1328.

17. 曾连开.耳穴贴压联合穴位贴敷应用于术后参试者呕吐的临床护理观察[J].中医临床研究,2020,12(24):83-84.

18. 计进伟.耳穴贴压治疗单纯性肥胖症 31 例[J].中医外治杂志,2020,29(04):3.

19. Wirz-Ridolfi, Andreas. “The History of Ear Acupuncture and Ear Cartography: Why Precise Mapping of Auricular Points Is Important.” Medical acupuncture vol. 31,3 (2019): 145-156.

20. 田薇,金鹏,张宇虹,李滨辛.耳穴贴压结合中医按摩对腹腔镜胆囊切除术后参试者疼痛的影响[J].中国民间疗法,2020,28(15):41-43.

21. 周晓燕,陈梦兰.耳穴压丸联合抗早颗粒对性早熟女童第二性征及性激素水平的影响[J].湖北中医杂志,2020,42(08):13-15.

22. 郭耀光,孙光伟,胡纪可,龙梅,杨玲.体针联合耳穴贴压对脑卒中后失眠的影响[J].四川中医,2020,38(07):195-198.

23. 马孟婕,张留巧,王晴,王向丽.耳穴贴压在围手术期护理中的应用现状研究[J].中国中医药现代远程教育,2020,18(13):162-164.

24. 杨莉,徐艳辉,王荣坤,余利波,姚宇.耳穴贴压治疗原发性 BPPV 复位成功后残余症状的疗效[J/OL].听力学及言语疾病杂志:1-3[2021-01-06].

25. Lin, Wei-Chun et al. “Use of an Ecological Momentary Assessment Application to Assess the Effects of Auricular Point Acupressure for Chronic Low Back Pain.” Computers, informatics, nursing : CIN vol. 37,5 (2019): 276-282.

26. 张华佳.耳穴贴压与督脉灸联合应用于心虚型失眠症参试者的效果分析[J].医学理论与实践,2020,33(11):1855-1856.

27. 李晶如,赵倩,史磊,李慧莹,郭梓鑫.PPH 术后耳穴压丸联合心理干预的镇痛效果观察[J].中国继续医学教育,2020,12(15):196-198.

28. 纪新尊,汤锡锋,吉晓天.耳穴贴压联合芬太尼透皮贴对胃癌姑息治疗参试者疼痛强度、生活质量的影响[J].上海针灸杂志,2020,39(05):536-540.
